# The kyphosis–lordosis difference parameter and its utility in understanding the pathogenesis of adolescent idiopathic scoliosis

**DOI:** 10.1186/s13104-022-06067-3

**Published:** 2022-05-15

**Authors:** Adrian Gardner, Fiona Berryman, Paul Pynsent

**Affiliations:** 1 The Royal Orthopaedic Hospital NHS Foundation Trust, Bristol Road South, Northfield, Birmingham, B31 2AP UK; 2 Institute of Clinical Sciences, University of Birmingham, Edgbaston, Birmingham, B15 2TT UK

**Keywords:** Kyphosis, Lordosis, Scoliosis, AIS, Sagittal, Spine

## Abstract

**Objective:**

The relationship of sagittal spinal shape in the pathogenesis of adolescent idiopathic scoliosis (AIS) is recognised. What is not clear is the relationship between the sagittal shape of those without scoliosis and the potential development of AIS, including the greater prevalence in females. The use of a new parameter, the kyphosis–lordosis (KL) difference, was developed to explore this further.

**Results:**

The KL difference was calculated for 117 males and 79 females over seven years with 831 measures made. For females, the KL difference, between the ages of 9 and 12 ½ years, decreases from 5° to nearly 0° until starting to climb again from the age of 14 years, back to 5° by the age of 16 ½ years. For males, there is a gradual decline from 9° at age 9 years to 5° at age 17 years. Both age and sex were statistically significant in the development of the parameter. When comparing to previously published data around the true, de-rotated, sagittal shape of the scoliotic spine, the KL difference has utility in explaining the female predominance in the prevalence of AIS. This adds to the weight of evidence behind understanding why AIS develops.

## Introduction

The development of the sagittal shape of the spine is a hypothesis for the development of adolescent idiopathic scoliosis (AIS) [1–4]. However, information on the development of the sagittal shape of the spine in adolescents without scoliosis is less well described [5]. Linking the development of the shape of the spine in those with, and without, AIS is critical to the understanding of why some children develop AIS.

Our group has described the development of kyphosis and lordosis in the developing spine without scoliosis in a longitudinal fashion over 5 years [6] using surface topography (Integrated Shape Imaging System 2- ISIS2 [7]). This showed that age was statistically significant for both kyphosis and lordosis and sex was only significant in the development of lordosis. A re-analysis of kyphosis and lordosis modelling was performed to investigate the relationship of kyphosis and lordosis, when viewed together as one parameter. This paper shows the development of this new parameter with age, between males and females, explaining the potential relevance to the pathogenesis of scoliosis.

## Main text

### Background and ethical considerations

This is a re-analysis of prospectively collected longitudinal data of spinal and back shape following two further years of data collection. Ethical approval was via the NRES committee West Midlands—South Birmingham (11/H1207/10). The investigation was of children without spinal deformity, imaged using the ISIS2 surface topography system over seven years. ISIS2 is a radiation free method of gaining 3D information of the shape of the back and spine, with the individual standing unsupported in a comfortable upright stance. The development of kyphosis and lordosis of this group after 5 years of measures has been reported [6]. ISIS2 measures kyphosis and lordosis using a technique analogous to the Cobb method [8]. Kyphosis is measured between the vertebra prominens (VP) and the point of inflection between the sagittal curves and lordosis is measured between the point of inflection and the sacrum. Both kyphosis and lordosis are measured in degrees.

### Methods

Using a larger data set, the modelling of the development of kyphosis and lordosis was re-examined as previously described [6]. The kyphosis–lordosis (KL) difference was calculated, subtracting the lordosis from the kyphosis of each individual. The kyphosis, lordosis and KL difference were analysed longitudinally against age with linear mixed effect modelling [9], allowing for repeated measures from the same individual over time, using the lme4 [9] and ggplot2 [10] packages in R [11]. Linear mixed effect models were produced examining the effects of age and sex in the KL difference parameter with a model of the fixed effects of age and sex and the random effect of repeat measures. To examine the effects of age or sex, a model without that parameter was produced and compared using an ANOVA method to the model with both parameters [12]. Significance was predefined as p < 0.05.Reviewing the Akaike information criterion [13], a linear analysis was chosen as more complicated non-linear models did not improve the behaviour of the model.

### Results

The group consisted of 117 males and 79 females. Over the seven year period there were 831 measures. Not all were measured at every occasion, although 166 were measured on three or more occasions, comprising 787 of the total.

The age range of all participants was between 9 and 17 years. When all measurements were analysed individually, the mean height was 1.57 m (SD 0.13 m, range 1.30 to 1.88 m) for males and 1.55 m (SD 0.12 m, range 1.18 to 1.84 m) for females and the body mass index (BMI) was 20.4 kg/m^2^ (SD 3.2 kg/m^2^, range 13.8 to 31.6 kg/m^2^) for males and 19.7 kg/m^2^ (SD 2.9 kg/m^2^, range 14.0 to 33.1 kg/m^2^) for females.

The participants were in the main Caucasian, with approximately 3% from a non-Caucasian heritage.

**Fig. 1:**
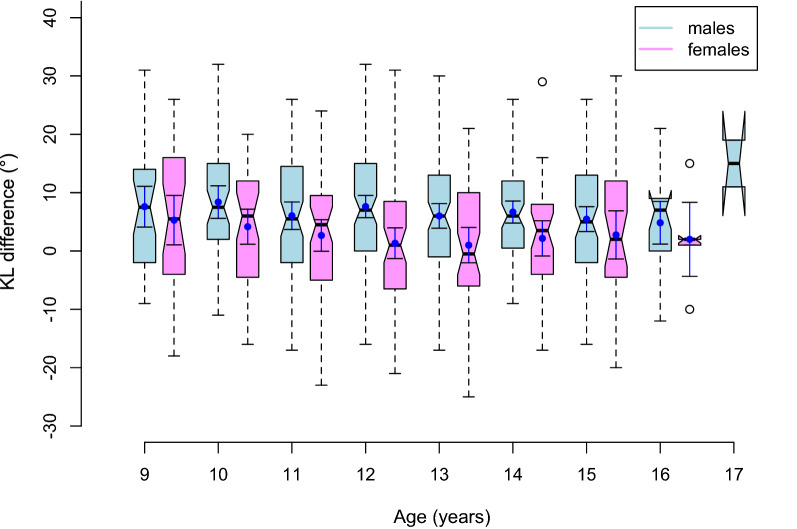
The KL difference (°) for males and females versus age (years). The thick horizontal line at the pinch point represents the median value with the box surrounding that, the interquartile range. The solid dark blue dot and associated bars represent the mean and 95% confidence interval for the mean. The dotted line and whiskers are up to 1.5 times the interquartile range and any data beyond that is seen as an open circle

**Fig. 2:**
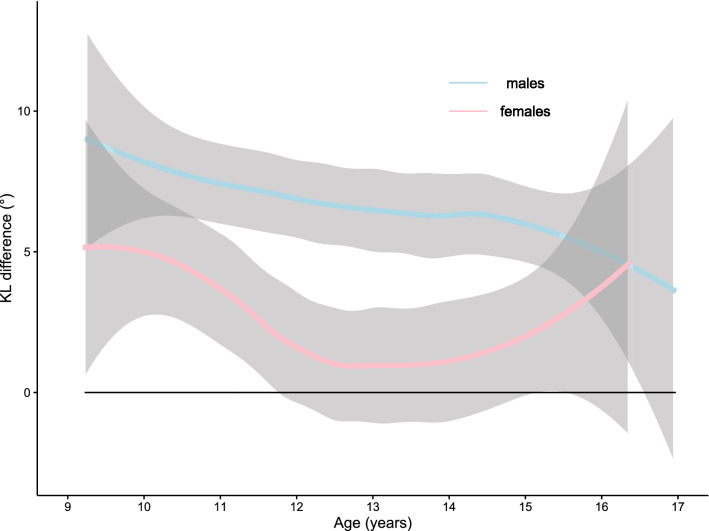
The KL difference loess lines only (°) with 95% confidence intervals for males and females versus age (years)

Figure 1 is a composite box and whisker plot of the KL difference against age. Figure 2 shows the KL difference in the form of loess lines with the 95% confidence interval as the grey funnel [14]. For females, the KL difference, between the ages of 9 and 12 ½ years, decreases from 5° to nearly 0° until climbing again from 14 years back to 5° by 16 ½ years. For males, there is a gradual decline from 9° at 9 years to 5° at 17 years. A decreasing KL value indicates a relative increase in lordosis versus kyphosis, or conversely, a decrease in kyphosis versus lordosis. The statistical analysis is shown in Table 1. For the KL difference, both age and sex were found to be statistically significant. The point of inflection was not found to be significant for kyphosis, lordosis or the KL difference.

**Table 1:** Statistical analysis of measured parameters against age and sex

	Age	Sex
Kyphosis	< 0.001	0.0648
Lordosis	< 0.001	0.009
Kyphosis Lordosis difference	0.016	< 0.001

### Discussion and conclusion

There is a paucity of understanding of the normal growth and development of children. Whilst growth standards are published for parameters that can be measured repeatedly without risk to the child (such as the WHO standards [15]), techniques that require the repeated use of ionising radiation have been avoided due to the risks [16]. Thus, serial radiographic measures of spinal shape during growth in those without spinal deformity do not exist. Other measures, such as ISIS2 surface topography (a radiation free technique), allow an understanding of normal growth and development.

AIS is a condition with a 3D deformation of the shape of the spine, seen as an alteration of shape away from the norm in the coronal, sagittal and axial planes, with the development of scoliosis, a change of kyphosis and lordosis and the development of intervertebral rotation [17]. There is growing literature supporting the hypothesis that AIS develops in humans and is not seen in other species because of the specific sagittal shape of the human spine [2, 3, 18], thought to occur because of the inter-species difference in pelvic shape and the development of lumbar lordosis [19].

AIS is more common in females [17]. The reasons for this are not clear. Previous research suggests there may be a link to the differences in the development of the sagittal profile of the spine between the sexes [5]. Our previous work did not show a statistical difference in the effect of sex on the development of kyphosis, though there was a difference in the shape of the curves [6]. There was a statistically significant difference with sex for the development of lumbar lordosis. However, linking the development the sagittal profile of the spine with the development of AIS [20] by considering only one segment of the spine in isolation would seem shortsighted. Previous literature in adult scoliosis [21] notes that the relationships between kyphosis and lordosis is related to clinical outcome, but nothing similar is in the AIS literature.

Consequently, the KL difference is a method that examines the magnitude of both the kyphosis and lordosis as a whole. The analysis presented here demonstrates that sex is highly statistically significant in the development of the KL difference. In females between the ages of 12 and 14, the decrease seen shows that the mean value is only just positive with the 95% confidence interval crossing zero. As seen in Fig. 2, there is a different shape to the plotted curves between males and females.

It has recently become possible to create and manipulate a 3D model of the spine using orthogonal radiographs from EOS imaging [22]. With a local coordinate system for each vertebral body, axial rotation and lateral bend from 3D models of the scoliotic spine can be excluded to reveal the true amount of kyphosis and lordosis, demonstrating that the true kyphosis from the 3D model is less than would be measured from a 2D radiograph of the same spine. When comparing the amount of kyphosis and lordosis from scoliotic 3D models with 3D models from non-scoliotic spines, there was less kyphosis in those with scoliosis compared to the non-scoliotics (non-scoliotics mean of 55° against a mean of 22° for thoracic curves and 34° for thoracolumbar curves) and more lordosis (non-scoliotics mean of 56° against a mean of 61° for thoracic curves and 65° for thoracolumbar curves). The KL difference parameter described here can be calculated using this data, with results of –2° for the non-scoliotics, –39° for thoracic curves and –31° for thoracolumbar curves, demonstrating the mismatch between kyphosis and lordosis in those with AIS. Of note, the values of kyphosis and lordosis quoted by Newton [22] are higher than previously reported [23, 24]. This is likely to reflect measurement differences between a 3D model and 2D measure from a radiograph, where the superior limit of measurement may not be the superior thoracic spine due to difficulties imaging through the shoulder girdle.

The lack of influence of the point of inflection in this analysis is expected given the work of Sanders [24] where, after the age of 9, the percentage contributions of the lengths of the thoracic and lumbar spines remain similar as age increases.

The results presented here add to the hypotheses describing the sagittal spinal shape as an initiating factor for the development of AIS [1–4]. It is the totality of the sagittal shape, rather than just the kyphosis or lordosis that creates the shape of the spine allowing for an overall spinal balance that is upright stance [25]. Consequently, the development with age of a situation where the relationship between kyphosis and lordosis changes will affect the overall sagittal shape, reflected by the changing KL difference. Given that the work of Newton [22] shows scoliosis to have a negative KL difference, and that the work presented here shows a difference between males and females with females having a smaller, and sometimes negative KL difference, there are similarities between the KL difference in a non-scoliotic and scoliotic population that may explain the sex distribution of AIS.

What this work does not do is differentiate between whether AIS is secondary to hypokyphosis, as popularised by Dickson [4], or to a posteriorly inclined segment over the thoracolumbar spine as described by Castelein [1, 18, 20] and others. It could be that the two hypothesis are complementary rather than exclusive given that, a relative lack of kyphosis, when compared to the lordosis in a spine, described by the KL difference, will likely increase the length of the posteriorly inclined segment. Further analysis of the sub-components of sagittal shape is beyond what is possible with these data but will form future work.

It is of note that the kyphosis–lordosis index is previously described as the internal dimensions of the thorax using an axial CT image [26]. The KL difference described in this paper is a different parameter describing sagittal spinal shape.

## Conclusion

This work presents the KL difference, a new parameter that describes how the overall sagittal shape of the spine in children without spinal deformity changes significantly with age and sex. This adds to the hypotheses around the development of the sagittal shape of the spine and the association to the development of AIS.

## Limitations

The weakness of ISIS2 is the inability to image individual vertebral bodies or the pelvis to quantify any changes in pelvic rotation occurring during the study. This would require serial radiographs in growing children, causing ethical issues such that it would never be performed. Also, this study did not analyse the effects of handedness, weight of school bags or BMI with regards the KL difference. Handedness has been found not to be significant in the development of kyphosis and lordosis separately [27] and is unlikely to be a factor in the KL difference. An increased BMI is known to increase kyphosis compared a normal BMI in AIS [28] so may have an effect on the KL difference. Carrying school bags alters spinal shape [29]. Whether that effect is prolonged after the weight is removed is not clear and so no supposition can be made for the effect of school bags on the KL difference.

## Data Availability

The data sets used and analysed during this study are available from the corresponding author on reasonable request.

## Abbreviations

AIS: Adolescence idiopathic scoliosis
KL difference: Kyphosis–lordosis difference
ISIS2: Integrated Shape Imaging System 2
NRES: National Research Ethics Service UK
3D: 3 Dimensional
VP: Vertebra prominens
ANOVA: Analysis of varience
SD: Standard deviation
BMI: Body mass index
WHO: World Health Organisation
2D: 2 Dimensional
